# Unwinding the tangle of adolescent pregnancy and socio-economic functioning: leveraging administrative data from Manitoba, Canada

**DOI:** 10.1186/s12884-023-05443-6

**Published:** 2023-03-04

**Authors:** Aleksandra Jakubowski, Leslie L. Roos, Elizabeth Wall-Wieler

**Affiliations:** 1 Department of Health Sciences, Department of Economics, Northeastern University, Boston, MA USA; 2 Department of Community Health Sciences, Max Rady College of Medicine, University of Manitoba, Room S113 - 750 Bannatyne Avenue, Winnipeg, MB R3E 0W3 Canada

**Keywords:** Adolescent pregnancy, High school graduation, Income assistance, Population-based cohort

## Abstract

**Background:**

Understanding the relationship between adolescent pregnancy and adult education and employment outcomes is complicated due to the endogeneity of fertility behaviors and socio-economic functioning. Studies exploring adolescent pregnancy have often relied on limited data to measure adolescent pregnancy (i.e. birth during adolescence or self-reports) and lack access to objective measures of school performance during childhood.

**Methods:**

We use rich administrative data from Manitoba, Canada, to assess women’s functioning during childhood (including pre-pregnancy academic performance), fertility behaviors during adolescence (live birth, abortion, pregnancy loss, or no history of pregnancy), and adult outcomes of high school completion and receipt of income assistance. This rich set of covariates allows calculating propensity score weights to help adjust for characteristics possibly predictive of adolescent pregnancy. We also explore which risk factors are associated with the study outcomes.

**Results:**

We assessed a cohort of 65,732 women, of whom 93.5% had no teen pregnancy, 3.8% had a live birth, 2.6% had abortion, and < 1% had a pregnancy loss. Women with a history of adolescent pregnancy were less likely to complete high school regardless of the outcome of that pregnancy. The probability of dropping out of high school was 7.5% for women with no history of adolescent pregnancy; after adjusting for individual, household, and neighborhood characteristics, the probability of dropping out of high school was 14.2 percentage points (pp) higher (95% CI 12.0-16.5) for women with live birth, 7.6 pp. higher (95% CI 1.5-13.7) for women with a pregnancy loss, and 6.9 pp. higher (95% CI 5.2-8.6) for women who had abortion. They key risk factors for never completing high school are poor or average school performance in 9th grade. Women who had a live births during adolescence were much more likely to receive income assistance than any other group in the sample. Aside from poor school performance, growing up in poor households and in poor neighborhoods were also highly predictive of receiving income assistance during adulthood.

**Discussion:**

The administrative data used in this study enabled us to assess the relationship between adolescent pregnancy and adult outcomes after controlling for a rich set of individual-, household-, and neighborhood-level characteristics. Adolescent pregnancy was associated with higher risk of never completing high school regardless of the pregnancy outcome. Receipt of income assistance was significantly higher for women having a live birth, but only marginally higher for those who had a pregnancy that ended in loss or termination, underlining the harsh economic consequences of caring for a child as a young mother. Our data suggest that interventions targeting young women with poor or average school marks may be especially effective public policy priorities.

**Supplementary Information:**

The online version contains supplementary material available at 10.1186/s12884-023-05443-6.

## Background

Adolescent pregnancy may pose an important impediment to young women’s education attainment and lifetime earning potential [1]. Understanding the impact of adolescent pregnancy on education and earnings is challenging due to endogeneity of fertility behaviors and socio-economic functioning [2]. Women who become pregnant in adolescence may have been predisposed to poor school performance, which in turn may make the prospect of starting a family early in life preferable to continuing education. Alternatively, women who become pregnant during adolescence may experience a disruption to their schoolwork [3], which affects their school performance, and thus their chances of completing high school and achieving higher earning potential [4, 5]. Adolescent pregnancy has also been documented to have negative consequences on education of future generations: women who have their first child in adolescence tend to have children and grandchildren who are less ready for school [6].

Existing research on adolescent pregnancy and motherhood has been stymied by limited data about the underlying characteristics of women that may explain both adolescent pregnancy and education/earning potential. Even some of the most comprehensive datasets lack objective data about the socio-economic functioning of the household in which a woman grew up, including history of teen pregnancies in the family, school performance during childhood (prior to adolescent pregnancy), and adolescent pregnancy status [7–9]. This study leverages robust administrative data tracking the socio-economic and health functioning of all residents of Manitoba, Canada, to test the association between socio-economic functioning of women during their childhoods, fertility behaviors during adolescence, and adult education and economic outcomes.

We contribute to the existing literature on adolescent pregnancy and adult education/economic outcomes by relying on objective measures of pregnancy, school performance, and socio-economic functioning. Since all residents of Canada are part of a universal healthcare system, we are able to use administrative records to determine adolescent pregnancy status (including whether the pregnancy resulted in live birth, stillbirth/miscarriage, or abortion) for each female resident of Manitoba born between 1982 and 1997. We then link each woman’s data on pregnancy with her individual, household, and neighborhood characteristics. Using this rich set of covariates, we calculate propensity score weights to help adjust for characteristics that may be predictive of adolescent pregnancy. We then compare the probability of dropping out of high school and using income assistance before age 20 among women who had a teen pregnancy versus women who did not, while controlling for characteristics from *before* the teen pregnancy. Finally, we examine whether risk factors associated with high school graduation and early adult income assistance differ for women with and without adolescent pregnancy.

## Methods

### Setting

The setting of this study is Manitoba, a central Canadian province with approximately 1.3 million residents [10]. In 2010, the percent of adolescent women who had a pregnancy was higher in Manitoba (4.8%) than in Canada (2.8%) as a whole; Manitoba also saw higher rates of pregnancy terminations among adolescent women (1.9%) than across Canada (1.5%) [11]. Through the universal health care insurance plan, all women in Manitoba have legal, safe, and free access to pregnancy termination in Manitoba; those aged <18 do not require consent from a parent or guardian to terminate their pregnancy [12]. Although abortions are legal, access to abortions in Manitoba is limited, particularly in rural areas [13], due to fewer services available in remote areas and long distances required to travel to obtain services [14]. are covered through insurance plans or paid for out-of-pocket; thus, cost can be a barrier to obtaining contraceptives. However, access to confidential low- or no-cost birth control and contraceptives are available through community health programs such as Klinic [15].Among Manitobans between 25 and 64 years old in 2016, 85.6% had finished high school or equivalent; this is slightly lower than the Canadian rate of 88.5% [16]. In 2018, approximately 5.3% of Manitobans were income assistance beneficiaries, which is similar to the overall Canadian rate of 5.1% [17, 18].

### Data

The Manitoba Population Research Data Repository contains province-wide, routinely collected individual data for each resident [19]. Here, we linked data from the Manitoba Health Insurance Registry with individual-level information from medical services and hospital abstracts (from Manitoba Health, Seniors, and Active Living), Social Assistance Management Information Network Research Data (from Manitoba Families), Enrollment, Marks, and Assessment (from Manitoba Education), and the Canadian Census [20–24]. An anonymized personal health number allowed linkage of these de-identified datasets. Information on linkage methods, confidentiality, privacy, and validity has been fully documented [25, 26].

### Cohort

**Fig. 1:**
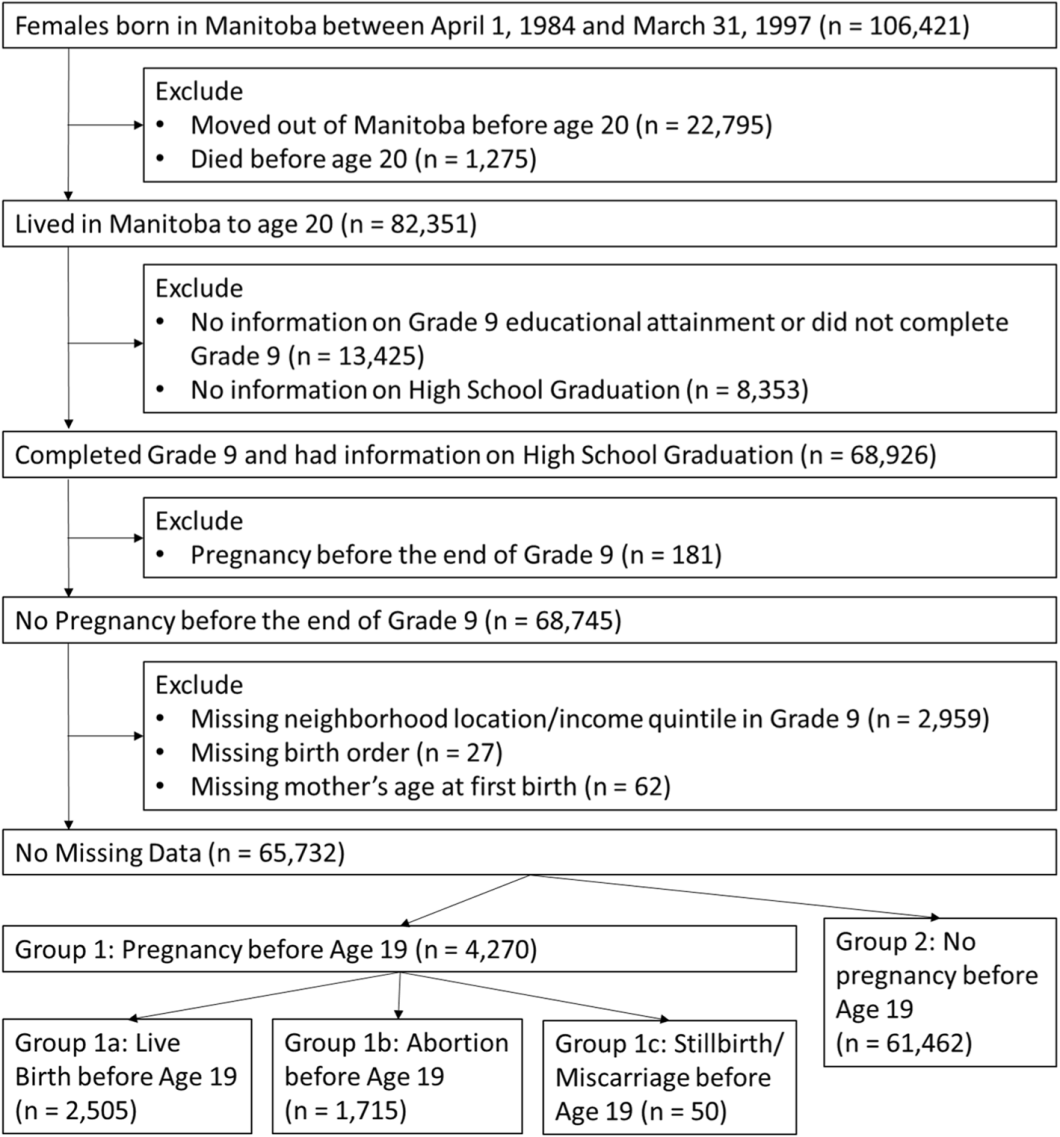
Cohort selection process

Our sample consisted of all women born in Manitoba between April 1, 1982 and March 31, 1997 who survived until age 20 or later and who were stable residents of Manitoba, i.e. lived in the province since birth. We excluded women who were missing information on relevant neighborhood, household, and individual variables. We also excluded pregnancies *before* age 14 or the 9th grade, since our study design relied on using pre-pregnancy data of individual, household and neighborhood functioning, which were measured using 9th grade data. Our final cohort included 65,732 women (Fig. 1) between the ages of 14 and 20.

### Adolescent pregnancy

We defined adolescent pregnancy as any pregnancy occurring before age 19. This definition is widely used in the existing literature [27]. Each woman was categorized into one of four adolescent pregnancy outcomes: no adolescent pregnancy, adolescent pregnancy that resulted in a live birth, adolescent pregnancy that resulted in a stillbirth or miscarriage, and adolescent pregnancy that was terminated by an abortion. For women having multiple pregnancies with at least one live birth and one stillbirth (< 1% of the sample), the pregnancy outcome was defined as a live birth. Each pregnancy outcome is defined using ICD-9 (before April 1, 2004) and ICD-10 (April 1, 2004 onward) codes from hospital discharge abstracts; see Supplemental Table 1 for a complete list of codes.

### Outcomes

Our two outcomes of interest are 1) did not complete high school (HS) and 2) received employment and income assistance (EIA) as a primary applicant before age 20. High school completion is defined as having achieved the required number of high school credits, or having an indication of high school graduation in their education records within six years of entering grade 9 [28]. In Manitoba, Employment Income Assistance (EIA) is available to individuals aged 18-65 whose monthly basic needs and housing costs exceed their financial resources; needs are assessed based on household composition (number of individuals, ages and relationship to each other) and costs of ongoing health needs [29]. Specific support is also available to single parents, which would be available to adolescent mothers without partners [30]. For example, in 2018, the total monthly tax-free income provided by EIA to a one-adult household with no children was $820 if the adult did not have a disability and $1036 if the adult had a disability [31, 32]; a one-adult household with one child under age 7 received $1832 and a two-adult household with one child under age 7 received $1913 [31, 33].

### Covariates

We include a range of individual, household, and neighborhood-level variables that could be related to adolescent pregnancy, early adult education, and income assistance. Individual-level variables include birth order (1, 2, 3, 4+), birth year cohort (1982-1985, 1986-1989, 1990-1993, 1994-1997), average grade on classes taken in 9th grade (<50%, 50-79%, 80-100%), and mental illness during Grade 9 year (including attention-deficit/hyperactivity disorder, conduct disorder, substance use disorder, mood and anxiety disorder, and psychosis). Household-level variables include mother’s age at first birth (<19, 19 or older), and whether her parent(s) received income assistance when she was in 9th grade. Neighborhood-level variables included income quintile (from 1 = lowest to 5 = highest income), location (urban/rural), and percent of residents aged 25-64 years who completed high school (<50, 50-74, 75-89, ≥ 90). Neighborhoods are defined by 6-digit postal code, and consist of 400 to 700 people. Data sources and definitions for each variable are shown in Supplemental Table S1.

### Statistical model

To estimate the association between adolescent pregnancy status and the socio-economic outcomes of 1) high school completion and 2) income assistance in early adulthood, we fit generalized estimating equations (GEE) regression models with logistic link and binomial distribution clustered at neighborhood level. We are interested in testing how individual vs household vs neighborhood factors affected probability of HS drop out and income assistance in the four study groups, so these covariates are introduced sequentially:

1 $${Y}_{ihn}=\alpha + \beta {\textrm{AdolescentPregnancy}}_i+ {X'}_i\gamma+{\upvarepsilon}_{ihn}$$

2 $${Y}_{ihn}=\alpha + \beta {\textrm{AdolescentPregnancy}}_i+ {X'}_i\gamma+ {Z'}_{ih} \delta+{\upvarepsilon}_{ihn}$$

3 $${Y}_{ihn}=\alpha + \beta {\textrm{AdolescentPregnancy}}_i+ {X'}_i\gamma+ {Z'}_{ih} \delta+ {V'}_{ihn}\theta + {\upvarepsilon}_{ihn}$$

4 $${Y}_{ihn}=\alpha + \beta {\textrm{AdolescentPregnancy}}_i+ {X'}_i\gamma+ {Z'}_{ih} \delta+ {V'}_{ihn}\theta + {\textrm{IPW}}_{\textrm{i}} + {\upvarepsilon}_{ihn}$$

where Y is the outcome variable (dropped out of high school, received income assistance before age 20) of woman *i* who lived in household *h* in neighborhood *n.* Adolescent pregnancy has four levels – no pregnancy (reference group), live birth, abortion, and stillbirth/miscarriage. X_*i*_ is a vector of individual-level characteristics of woman *i* (birth order, birth year, grade 9 school achievement, mental illness). Z_h_ is a vector of household-level covariates (mother had first child during adolescence, family received income assistance), and V_n_ is a vector of neighborhood-level covariates (income quantile, rural or urban location, high school completion rate). IPW_i_ is the inverse probability of adolescent pregnancy weight, obtained from a logistic regression model including all individual, household, and neighborhood covariates. e_*ihn*_ is the stochastic error term [34].

Given the difficulty of interpreting logistic regression coefficients (log odds) and exponentiated coefficients (odds ratios), we instead report average marginal effects (ME). Marginal effects represent the percentage point change in predicted probability of the outcome for a one unit increase of the independent variable (or percentage point change from base for discrete variables), holding all other variables constant.

Next, we examine which risk factors are associated with the probability of HS drop-out and receipt of income assistance among women having a teenage pregnancy (live birth, stillbirth, miscarriage, or abortion) and women not having a teenage pregnancy. In other words, we fit the GEE model specified in Eq. 5 separately for women with and without history of adolescent pregnancy:

5 $${Y}_{ihn}=\alpha + {X'}_i\gamma+ {Z'}_{ih} \delta+ {V'}_{ihn}\theta + {\textrm{IPW}}_{\textrm{i}} + {\upvarepsilon}_{ihn}$$

where the outcome variables and the model covariates are as defined  in Eqs. 1-4. All data management, programming, and analyses were conducted using SAS version 9.4 (Cary, NC). Data visualization was done in R.

This study was approved by the Health Research Ethics Board at the University of Manitoba (#H2019:110) and the Health Information Privacy Commission at Manitoba Health, Seniors and Active Living (#2018/2019-70). Using de-identified administrative data files did not require obtaining informed consent from participants.

## Results

Our cohort included 65,732 women, the vast majority of whom (93.5%) had no pregnancy during adolescence. Of the 4270 women who were pregnant between ages 14 and 19, 58.7% had a live birth, 40.2% had abortion, and 1.2% had a stillbirth or miscarriage. Figure 1 summarizes the composition of our sample and the cohort selection procedure.

Ninth grade school performance, prior to any pregnancy recorded in our data, was much higher among women without a teen pregnancy compared to women with a teen pregnancy, regardless of pregnancy outcome (Table 1). For example, 64% of women without adolescent pregnancy earned top marks in 9th grade compared to just 12% of women who had a live birth, 24% of women who had an abortion, and 14% of women who had a stillbirth or miscarriage. Women who became  pregnant during adolescence were more likely to live in poor households and have a mother who became pregnant as an adolescent. Approximately 60% of women who had no adolescent pregnancy lived in the top two neighborhood wealth quintiles compared to 63% of women who had a live birth residing in the lowest two neighborhood wealth quintiles. Women who had an abortion during adolescence lived mostly in urban areas and were equally split across the 5 wealth quintiles.

**Table 1:** Characteristic of Cohort, by Adolescent Pregnancy Status (*n* = 65, 732)

Characteristics	No Pregnancy in Adolescence (*n* = 61,462)	Adolescent Pregnancy (*n* = 4270)		
		Live Birth (*n* = 2505)	Abortion (*n* = 1715)	Stillbirth/ miscarriage (*n* = 50)
	N (%)	N (%)	N (%)	N (%)
**Individual**				
Birth Order				
1	26,693 (43.4)	962 (38.4)	759 (44.3)	19 (38.0)
2	22,006 (35.8)	780 (31.1)	614 (35.8)	18 (36.0)
3	9034 (14.7)	467 (18.6)	244 (14.2)	<6
4+	3729 (6.1)	296 (11.8)	98 (5.7)	Suppressed
Birth Year				
1984-1985	7775 (12.7)	388 (15.5)	335 (19.5)	11 (22.0)
1986-1989	18,554 (30.2)	856 (34.1)	649 (37.8)	11 (22.0)
1990-1993	19,222 (31.3)	905 (36.1)	549 (32.0)	22 (44.0)
1994-1997	15,911 (25.9)	357 (14.2)	182 (10.6)	6 (12.0)
Grade 9 Achievement				
Top grades: 80-100%	39,113 (63.6)	302 (12.1)	415 (24.2)	7 (14.0)
Average Grades: 50-79%	21,025 (34.2)	1638 (65.4)	1148 (66.9)	34 (68.0)
Poor Grades: < 50%	1324 (2.2)	565 (22.6)	152 (8.9)	9 (18.0)
Mental Illness during Grade 9 year^a^	9458 (15.4)	663 (26.5)	391 (22.8)	14 (28.0)
**Household**				
Parent(s) received income assistance during Grade 9 year	2503 (4.1)	556 (22.2)	197 (11.5)	11 (22.0)
Mother was adolescent mom	5971 (9.7)	972 (38.8)	395 (23.0)	19 (38.0)
**Neighborhood at entry into Grade 9**				
Income quintile				
1 – Lowest Income	7362 (12.0)	938 (37.5)	337 (19.7)	14 (28.0)
2	10,503 (17.1)	576 (23.0)	386 (22.5)	14 (28.0)
3	12,504 (20.3)	423 (16.9)	331 (19.3)	7 (14.0)
4	14,424 (23.5)	343 (13.7)	365 (21.3)	8 (16.0)
5 – Highest Income	16,669 (27.1)	225 (9.0)	296 (17.3)	7 (14.0)
Location				
Urban	36,303 (59.1)	1338 (53.4)	1226 (71.5)	30 (60.0)
Rural	25,159 (40.9)	1167 (46.6)	489 (28.5)	20 (40.0)
Percent of residents aged 25-64 who completed High School				
< 50	3284 (5.34)	451 (18.0)	105 (6.1)	Suppressed
50-74	21,183 (34.5)	1132 (45.2)	751 (43.8)	12 (24.0)
75-90	27,495 (44.7)	813 (32.5)	695 (40.5)	29 (58.0)
≥ 90	9500 (15.5)	109 (4.4)	164 (9.6)	<6

*Note*: Cells with <6 suppressed; if only one category is <6, an additional cell is also suppressed to ensure cell number cannot be calculated

^a^Diagnoses include ADHD, Conduct Disorder, Substance Use Disorder, Mood and Anxiety Disorders, Psychosis

Relatively low proportion of women with no history of adolescent pregnancy dropped out of high school (8%) or received income assistance (3%) by age 20, compared to more than half of women who had a live birth dropping out of high school (53%) and receiving income assistance (55%) (Table 2). A high proportion of women who had a stillbirth or miscarriage also dropped out of high school (42%) but much fewer received income assistance (24%). About a third of women (28%) who had an abortion dropped out of high school and 12% received income assistance.

**Table 2:** Probability of Dropping out of High School and Receiving Income Assistance, by Adolescent Pregnancy Status

Adolescent Pregnancy Status	Dropped out of High School (95% CI)		Received Income Assistance (95% CI)	
	Crude	Adjusted^a^	Crude	Adjusted^a^
No Adolescent Pregnancy	0.08 (0.06, 0.09)	0.09 (0.08, 0.10)	0.03 (0.03, 0.04)	0.04 (0.03, 0.04)
Live birth	0.53 (0.48, 0.58)	0.23 (0.21, 0.25)	0.55 (0.48, 0.63)	0.26 (0.22, 0.30)
Still birth/Miscarriage	0.42 (0.31, 0.53)	0.16 (0.10, 0.22)	0.24 (0.13, 0.35)	0.08 (0.03, 0.13)
Abortion	0.28 (0.25, 0.30)	0.16 (0.14, 0.18)	0.12 (0.09, 0.14)	0.06 (0.05, 0.07)

^a^adjusted for individual, household, and neighborhood characteristics

**Fig. 2:**
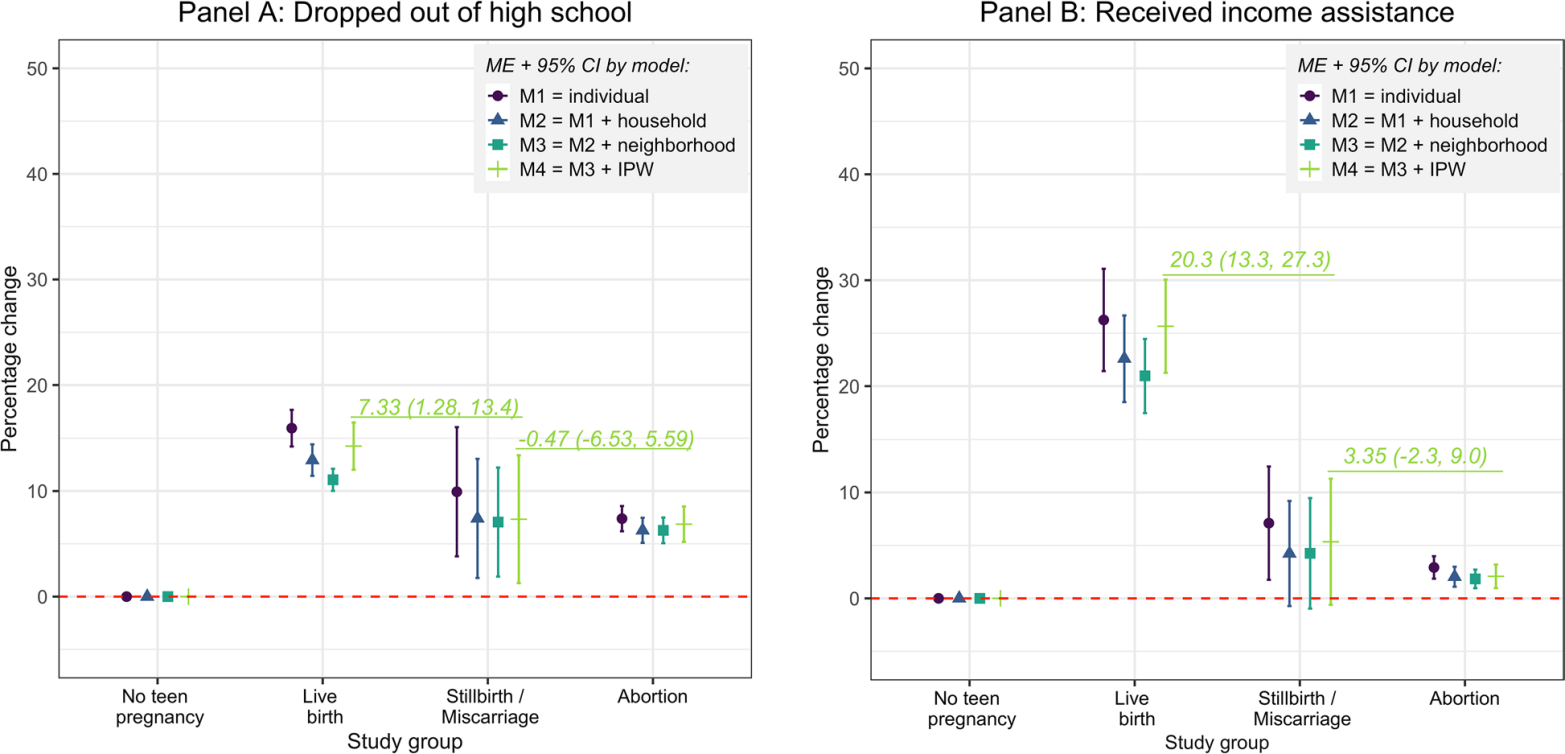
Change in probabilities of dropping out of high school and receiving income assistance reported by women with no teen pregnancy compared with those experiencing teen pregnancy. * Notes: Figure shows marginal effects (ME) and 95% confidence intervals (CI) calculated based on four GEE models with binary distribution, binomial link, and exchangeable correlation. Model 1 (M1) regressed the teen pregnancy indicators (none, live birth, stillbirth/miscarriage, abortion) on binary outcome variables of dropping out of high school (HS) and receiving income assistance (IA) adjusted for individual-level covariates (age at first pregnancy, 9th grade school performance, birth year, birth order). Model 2 (M2) added household-level covariates (indicator of whether the participant’s mother was a teen mother, indicator of receiving income assistance before high school). Model 3 (M3) added neighborhood-level covariates (average income of people in the neighborhood (in quintiles), urban/rural location, proportion of neighborhood graduated high school). Model 4 (M4) included the fully-adjusted models plus inverse probability weights (IPW) of teen pregnancy calculated based on all covariates stated above. We also ran postestimation Wald tests for equivalency of MEs between key study groups. In Panel A, we show the change in probability of dropping out of high school between Live birth vs. Stillbirth/Miscarriage groups (7.33pp, 95% CI: 1.28, 13.4) and Stillbirth/Miscarriage vs. Abortion groups (0.47pp, 95% CI: -6.53, 5.59). In Panel B, we show the probability change of receiving income assistance between Live birth vs. Stillbirth/Miscarriage groups (20.3, 95% CI: 13.3, 27.3) and Stillbirth/Miscarriage vs. Abortion groups (3.35, 95% CI: -2.30, 9.0)

Figure 2 summarizes our main findings, or the probability of adult socio-economic outcomes (dropping out of high school and income assistance), comparing the reference group of women without a teen pregnancy to those experiencing a teen pregnancy. We present four models, each with an additional set of covariates. For simplicity, we focus the presentation of results to the fully adjusted model with inverse probability weights (M4).

Women who had an adolescent pregnancy were much more likely to drop out of high school compared to women without an adolescent pregnancy, regardless of the pregnancy outcome (Fig. 2, Panel A). Probability of dropping out of high school was 14.2 percentage points (pp) higher (95% CI 12.0-16.5) for women with live birth, 7.6 pp. higher (95% CI 1.5-13.7) for women with miscarriage, and 6.9 pp. higher (95% CI 5.2-8.6) for women who had abortion. The risk of dropping out of high school was 7.33 pp. higher for women with live birth than for women with miscarriage (95%CI 1.28, 13.4) or women who had abortion. Risk of not completing high school was approximately equal for women who had miscarriage vs. those who had abortion (0.47pp (−6.53, 5.59)).

Women who had a teen pregnancy, particularly those who had a live birth, were much more likely to receive income assistance by age 20 compared to women without a teen pregnancy (Fig. 2, Panel B). Probability of receiving income assistance was 25.8 pp. higher (95% CI 21.6-30.0) for women with live birth, 5.7 pp. higher (95% CI -0.3-11.7) for women with miscarriage, and 2.1 pp. higher (95% CI 1.0-3.2) for women who had abortion. Women who had a live birth during adolescence were much more likely to receive income assistance than any other group.

**Fig. 3:**
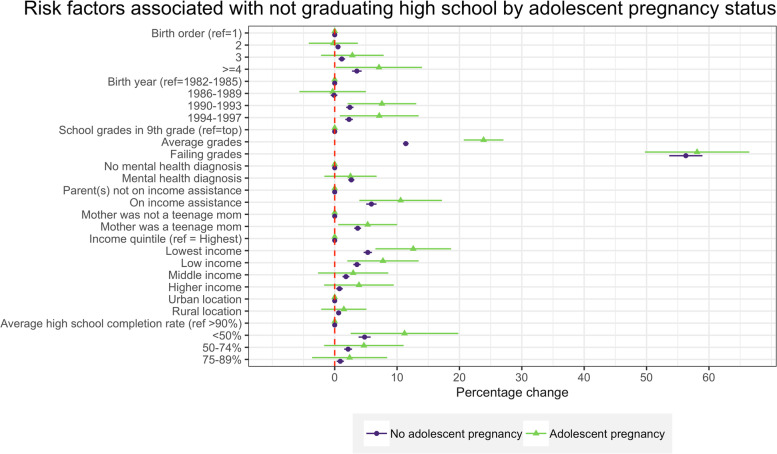
Risk factors associated with dropping out of high school, separately for women with and without teen pregnancy. Notes: Figure displays marginal effects (ME) and 95% confidence intervals (CI) calculated based on GEE models with binary distribution, binomial link, and exchangeable correlation. Models were adjusted for individual-level, household-level, and neighborhood-level characteristics and included inverse probability weights. Models were stratified by teen pregnancy status: 1) women who experienced teen pregnancy regardless of pregnancy outcome (live birth, stillbirth, miscarriage, and abortion) and 2) women who had no history of teen pregnancy

Some risk factors were more predictive of HS drop out for women with adolescent pregnancy than for women without teen pregnancy, though largely the differences were not statistically significant (Fig. 3). For example, risk factors such as living in a low-income household, and in a poor, less educated neighborhood were associated with a higher risk of high school dropout for women with adolescent pregnancy. Average school performance was associated with higher risk of high school dropout for all women, but the effect size was larger for women with adolescent pregnancy (23.7 pp. increase, 95% CI 20.5-26.9) than women without teen pregnancy (11.3 pp. increase, 95% CI 10.9-11.8). Poor school performance in 9th grade was by far the highest risk factor for dropping out of high school for all women: 57.4 pp. increase (95% CI 0.49-65.9) for women with adolescent pregnancy and 55.8 pp. increase (95% CI 53.1-58.5) for women without adolescent pregnancy.

**Fig. 4:**
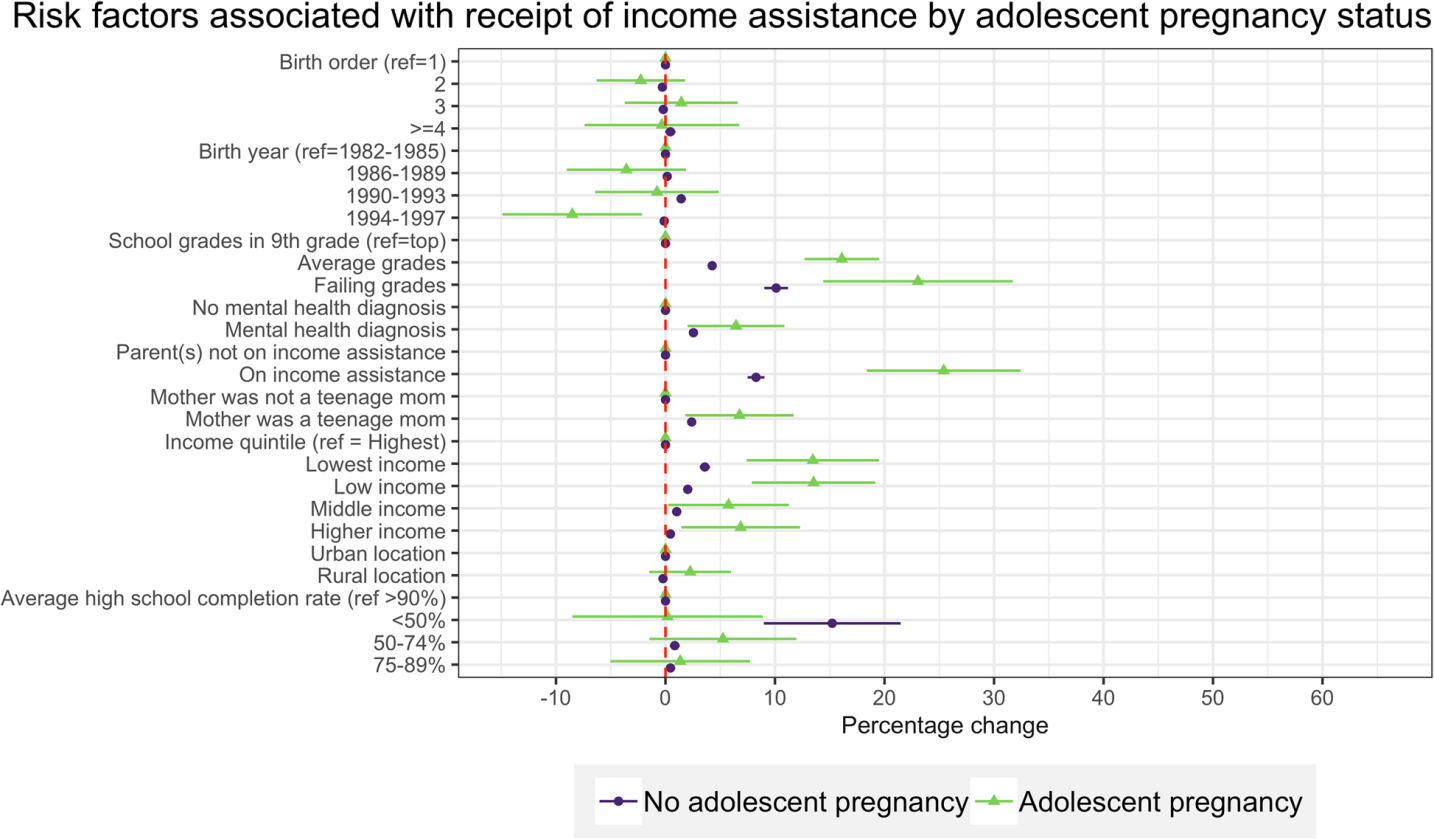
Risk factors associated with income assistance, separately for women with and without teen pregnancy. Notes: Figure displays marginal effects (ME) and 95% confidence intervals (CI) calculated based on GEE models with binary distribution, binomial link, and exchangeable correlation. Models were adjusted for individual-level, household-level, and neighborhood-level characteristics and included inverse probability weights. Models were stratified by teen pregnancy status: 1) women who experienced teen pregnancy regardless of pregnancy outcome (live birth, stillbirth, miscarriage, and abortion) and 2) women who had no history of teen pregnancy

Multiple factors were significantly associated with an increased risk of receiving income assistance among women having an adolescent pregnancy (Fig. 4). Poor school performance in 9th grade, living in poor households, and residing in impoverished neighborhoods were the risk factors most associated with needing income assistance in adulthood. Living in a household that received income assistance in 9th grade was associated with 25.6 pp. increased risk (95% CI 18.6-32.6) of receiving income assistance for women with adolescent pregnancy compared to 8.6 pp. (95% CI 7.8-9.) for women who did not have an adolescent pregnancy. Other risk factors associated with income assistance included mental health diagnosis and being born to a teenage mom.

## Discussion

We use administrative data from Manitoba, Canada, to trace how childhood socio-economic functioning and fertility behaviors during adolescence affect women’s educational and economic outcomes in adulthood. Women who became pregnant during adolescence were significantly more likely to drop out of high school and receive income assistance compared to women who never had an adolescent pregnancy. Women whose pregnancy resulted in a live birth were most disadvantaged: the probability of dropping out of high school was 14.2 percentage points higher and the probability of receiving income assistance by age 20 was 25.8 percentage points higher than the reference women without teen pregnancy. Notably, young women who became pregnant during adolescence but either terminated their pregnancy or experienced a pregnancy loss, were more similar to the no pregnancy group than the live birth group. This likely reflects the large burden and stress of caring for a child during adolescence, which puts women at significant need of receiving income assistance in adulthood.

Perhaps not surprisingly, poor school performance was by far the most highly predictive risk factor for not completing high school, regardless of adolescent pregnancy status. The association between *average* school performance and probability of high school completion, however, diverged sharply by adolescent pregnancy status. The risk of dropping out of high school doubled for average school performers who had adolescent pregnancy (23.9 pp. increased risk) compared to average school performers who did not become pregnant as teens (11.4 pp. increase). Similarly, the association of poor and average school performance and receipt of income assistance was magnified for women who had teen pregnancy compared to those who did not. This suggests that for women who are at the margin of completing high school due to their school performance, the additional stress of adolescent pregnancy, and possibly their preference for starting a family rather than remain in school, may play a large role in whether they stay in school.

Among the key strengths of our study is access to the rich administrative data that enabled reconstruction of women’s socio-economic functioning during their childhood at the individual, household, and neighborhood levels. Access to records from the universal healthcare system enabled us to study adolescent pregnancy regardless of whether it resulted in live birth or not. The objective measures of education achievement in 9th grade as well as official records of high-school completion enabled us to avoid issues related to recall and social desirability bias of self-reported school performance data, which are primarily relied upon in similar research. Finally, our data enabled measuring not only individual-level functioning of the women, but also objective and contemporaneous measures of the household and neighborhood circumstances in which they grew up.

Although the individual-level characteristics of women are highly influential to their lifetime trajectories, we find that the household-level and neighborhood-level risk factors are also closely related to poor socio-economic outcomes in adulthood. Particularly, poverty experienced at the household and neighborhood levels are passed down through the generations. Consistent with existing literature, we find that growing up in a household that was receiving income assistance and living in the poorest neighborhoods puts young women at risk for never finishing high school and becoming income recipients themselves in adulthood [6, 9]. Our findings add to this literature by showing that child bearing exacerbates the poverty trap, putting women who were growing up poor at even greater risk for needing income assistance in the future.

Our study was subject to several limitations. Although we used robust data, the non-experimental design of our study still limits our ability to make causal conclusions. We attempt to address the issue of endogeneity by assessing young women’s socio-economic functioning prior to the adolescence period when they may have experienced pregnancy. We also weighted our models by the inverse probability of adolescent pregnancy to adjust for the possible bias in our models. Our sample included a cohort of all stable residents of Manitoba born between 1982-1997, or nearly 66,000 women. Yet, since only about 6% of the sample had an adolescent pregnancy, our sample size was fairly limited, especially when we examined women separately based on the pregnancy outcome. Most notably, only 50 women in the sample had a pregnancy loss recorded during adolescence, and our conclusions about these women should be taken with caution. We believe that presenting the outcomes of this group is nonetheless important, since they represent a group of women whose fertility behaviors are likely most similar to the live birth group. Approximately 10% of the women in our sample were excluded from analysis due to missing data on high school graduation. Although it is possible that those women who were missing graduation data were more likely to not finish high school, given the large sample size there were still many individuals in each category and thus the estimates would likely not have been impacted by the exclusion of these individuals. Finally, though using administrative data strengthened our analysis in important ways, it also means that we relied on health system data to define teen pregnancy status. It is possible that some women who had a miscarriage so early that they did not need medical intervention may be misclassified into the “no teen pregnancy” group. We are reassured by the fact that if such misclassification exists, our results would be biased toward the null (i.e. the association between adolescent pregnancy and study outcomes would be under-estimates). Moreover, using objective administrative data to define teen pregnancy is less problematic than self-reported data, which has been widely documented to suffer from social desirability bias, particularly when measuring socially sensitive topics [35].

## Conclusions

Our study is unique in its ability to use objective data to measure not only whether an adolescent pregnancy has occurred, but also the outcome of that pregnancy (since all abortions, pregnancy losses, and live births are recorded in the same government dataset). We also have access to objective measures of women’s school performance in 9th grade, before any pregnancies were observed in the data, and characteristics related to the socio-economic conditions in which the women grew up. We find lower rates of high school completion among women who had an adolescent pregnancy, regardless of the pregnancy outcome, suggesting that factors that contribute to teen pregnancy, but not necessarily teen parenthood, play an important role in the decision to finish secondary school. The use of income assistance, on the other hand, is significantly higher for women experiencing a live birth but only marginally higher for those whose pregnancy ended in loss or abortion; the burden of caring for a child as a young mother appears to have harsh economic consequences on women. Among the most predictive risk factors of never completing high school and receiving income assistance was poor and average school performance. Interventions that prioritize young women who struggle with their studies and focus on ensuring access to high quality reproductive health education and services, including contraceptive methods, may be especially effective public policy priorities.

## Supplementary Information

**Additional file 1: Table S1.** Definitions of variables, and correspond data sources.

## Data Availability

The datasets generated and analyzed for this study are not publicly available because of Manitoba privacy regulations for highly sensitive personal information data. Data are however available from the authors upon reasonable request and with permission from the University of Manitoba Health Research Ethics Board, the Health Information Privacy Commission at Manitoba Health, Seniors and Active Living, Manitoba Education, and Manitoba Families. Please contact Dr. Elizabeth Wall-Wieler (Elizabeth.Wall-Wieler@umanitoba.ca) for more information on the process for requesting the de-identified data used in this study.
