# Supplementary material for: Unwinding the tangle of adolescent pregnancy and socio-economic functioning: leveraging administrative data from Manitoba, Canada
Source: BMC Pregnancy Childbirth. 2023 Mar 4;23:140. doi: 10.1186/s12884-023-05443-6 (PMC9985199; doi:10.1186/s12884-023-05443-6)
Supplement: Supplementary file 1 — Additional file 1: Table S1. Definitions of variables, and correspond data sources. [file 12884_2023_5443_MOESM1_ESM.docx]

**Table S1.** Definitions of Variables, and correspond Data Sources

| **Variable** | **Definition** | **Data Sources** |
| --- | --- | --- |
| Adolescent Pregnancy (live birth, miscarriage/stillbirth, abortion) | Any pregnancy ending in a live birth, miscarriage, stillbirth, or abortion between the end of Grade 9 and 19^th^ birthday, defined using diagnoses codes (ICD-9-CM before April 1, 2004, ICD-10-CA starting on April 1, 2004) and procedure codes (ICD-9-CM before April 1, 2004, CCI starting on April 1, 2004).^1^   - Live Birth: ICD-9-CM diagnosis code V27, ICD-10-CA diagnosis code Z37, CCI codes 5.MD.5, 5.MD.60 - Miscarriage/Stillbirth: ICD-9-CM diagnosis codes 632, 633, 656.4, ICD-10-CA diagnosis codes O00, O02.1, O36.4, ICD-9-CM procedure codes 66.62, 75.0, CCI procedure code 5.CA.93 - Abortion: ICD-9-CM diagnosis codes 635, 636, ICD-10-CM diagnosis codes O03-O07, ICD-9-CM procedure codes 69.01, 69.51, 74.91, 75.0, CCI procedure codes 5.CA.88, 5.CA.89, 5.CA.90. | Hospital Abstracts |
| Birth Order | Whether she was first, second, third, or fourth or higher order birth among births to biological mother. | Manitoba Health Insurance Registry |
| Birth Year | Year of birth, divided into four groups: 1982-1983, 1984-1989, 1990-1993, 1994-1997 | Manitoba Health Insurance Registry |
| Average grade on classes taken in 9^th^ grade | The average of all classes taken during grade 9 school year, regardless of number of classes completed.^2^ Divided into three categories: <50%, 50-79%, 80-100% | Enrollment, Marks, and Assessments |
| In Foster Care during Grade 9 Year | Was in out-of-home care by Child and Family Services at some point between the beginning and end of grade 9 school year. | Child and Family Services Information System |
| Mental Illness during Grade 9 Year | Had at least one diagnosis during a hospitalization or physician visit with an ICD-9-CM or ICD-10-CA diagnosis code for at least one of the following mental health conditions during the grade 9 school year:   - Attention-deficit/hyperactivity disorder^3^: ICD-9-CM code 314; ICD-10-CA codes F90 - Conduct disorder^4^: ICD-9-CM code 312; ICD-10-CA codes F91 (except F91.3) - Substance use disorder^5^: ICD-9-CM codes 291, 292, 303, 304, 305; ICD-10-CA codes F10-F19, F55 - Mood and anxiety disorder^6^: ICD-9-CM codes 296.1-296.8, 300, 309, 311; ICD-10-CA codes F31-F33, F34.1, F38.0, F38.1, F40, F41, F42, F43.1, F43.2, F43.8, F44, F45.1, F45.2, F48, F68.0, F99, F53.0, F93.0 - Psychosis:^7^ ICD-9-CM codes 295, 297, 298; ICD-10-CA codes F11.5, F12.5, F13.5, F14.5, F16.5, F17.5, F18.5, F19.5, F20, F22, F23, F24, F25, F28, F29 | Hospital Abstracts, Medical Services |
| Mother’s age at First Birth | The age of biological mother when she had her first live birth. | Manitoba Health Insurance Registry |
| Parent(s) received income assistance in 9^th^ grade | Was the dependent of a person who received income assistance for at least one month between the beginning and end of grade 9 school year. | Social Assistance Management Information Network Research Data Set |
| Neighborhood (at Grade 9 entry) Income Quintile | Dissemination area-level average household income values from the census are used to construct neighborhood income quintiles. The income quintile of the neighborhood is defined by methods developed at MCHP and are ranked from 1 (lowest income) to 5 (highest income); these quintiles are created separately for rural and urban Manitoba.^8^ | Manitoba Health Insurance Registry/ Canadian Census |
| Percent of residents aged 25-64 years that completed high school | Dissemination area-level high school completion rate, among those living in the dissemination are aged 25-64, based on values from the census. | Manitoba Health Insurance Registry/ Canadian Census |

**References**

1. Manitoba Centre for Health Policy. Concept: Teenage pregnancy. http://mchp-appserv.cpe.umanitoba.ca/viewConcept.php?conceptID=1248. Published 2013. Accessed November 26, 2015.

2. Manitoba Centre for Health Policy. Concept: Grade 9 Achievement Index. Concept Dictionary and Glossary. http://mchp-appserv.cpe.umanitoba.ca/viewConcept.php?printer=Y&conceptID=1368. Published 2019. Accessed May 8, 2020.

3. Manitoba Centre for Health Policy. Concept: Attention-Deficit Hyperactivity Disorder (ADHD).

4. Manitoba Centre for Health Policy. Concept: Conduct Disorder. http://mchp-appserv.cpe.umanitoba.ca/viewConcept.php?conceptID=1449. Published 2016. Accessed November 2, 2017.

5. Manitoba Centre for Health Policy. Concept: Substance Use Disorder/Substance Abuse - Measuring Prevalence. http://mchp-appserv.cpe.umanitoba.ca/viewConcept.php?conceptID=1471. Published 2016. Accessed September 26, 2016.

6. Manitoba Centre for Health Policy. Concept: Mood and Anxiety Disorders - Measuring prevalence. Manitoba Centre for Health Policy. http://mchp-appserv.cpe.umanitoba.ca/viewConcept.php?conceptID=1391. Published 2015. Accessed January 30, 2016.

7. Manitoba Centre for Health Policy. Concept: Psychotic Disorders - Measuring Prevalence. Concept Dictionary and Glossary.

8. Manitoba Centre for Health Policy. Concept: Income quintile ranking procedure. Manitoba Centre for Health Policy. http://mchp-appserv.cpe.umanitoba.ca/viewConcept.php?printer=Y&conceptID=1164. Published 2002. Accessed February 15, 2016.
